# Bridging the gap in nocardiosis treatment: the promise of nanoparticle-based antimicrobials

**DOI:** 10.1097/MS9.0000000000004330

**Published:** 2025-11-24

**Authors:** Hoorain Akhtar, Sumera Ayoub, Arsalan Khan, Muhammad Talha, Mahnoor Fatima

**Affiliations:** a Department of Medicine, Loralai Medical College, Loralai, Pakistan; b Department of Medicine, King Edward Medical University, Lahore, Pakistan; c Department of Medicine, Shaheed Ziaur Rahman Medical College, Rajshahi Medical University, Bogura, Bangladesh

*To the Editor,*

*Nocardia* is a group of gram-positive bacteria mainly found in contaminated soil and water. The portal of entry includes inoculation, inhalation, or skin injury. *Nocardia* infection is a type of opportunistic infection (OI). It may cause pneumonia, brain abscess, and cutaneous nocardiosis. A recent taxonomic study discovered 119 species of *Nocardia*, among them 54 were linked with human infections. Some of which include *N. nova, N. brasiliensis, N. farcinica*, and *N. abscessus*. Each *Nocardia* species is different in its antibiotic susceptibility patterns, and its distribution diversifies geographically^[1]^. *Nocardia* affects mainly immunocompromised patients, who have undergone organ transplant and/or treated with corticosteroids, and Chronic Obstructive Pulmonary Disease (COPD) patients, and causes lesions in the lungs (i.e., Bronchiectasis). Nocardiosis treatment requires a long-term combination regimen^[1]^. This is in line with the TITAN Guidelines on the need for transparency in AI use in health care^[2]^.

*Nocardia* species are resistant to multiple drugs. In a study, an 85% success rate was found when patients with nocardiosis were treated with an antibiotic, i.e., Trimethorprim-sulfamethoxazole (TMP-SMX) alone or in combination with many drugs, such as linezolid, amikacin, or imipenem, and was effectively cured. In some other studies, 5.4% of *Nocardia* species showed growing resistance toward TMP-SMX; 42% of species were insusceptible to TMP-SMX; values differ due to different geographical regions^[1]^. Each year, at least 50,000 people die due to antimicrobial drug resistance worldwide. Using antimicrobial-based nanoparticles is a good alternative for treating nocardiosis as it is one of the innovative, short-term, and most effective ways to reduce antimicrobial resistance^[3]^.

Nanoparticles have unique properties, such as small size with dimensions between 1 and 100 nm, a high surface-to-volume ratio, therefore beneficial for pharmacokinetics. These can be derived from many materials such as metals, metal oxides, liposomes, carbon, and quantum dots^[4]^. Nanoparticles disrupt bacterial cell membranes, generate reactive oxygen species, and prevent medication from being carried away as these particles adhere to bacterial membranes. Nanoparticles have multiple advantages in accomplishing a precise and localized delivery of antimicrobial compounds, consequently enhancing therapeutic efficacy^[5]^.

Nanoparticles are being used in many clinical trials, such as Arikace, a liposomal formulation of amikacin for treating Multi Drug Resistance (MDR) gram-negative bacteria; Linhaliq and Lipoquin, a liposomal formulation of ciprofloxacin for treating non-cystic fibrosis bronchiectasis patients with chronic lung infections. Although nanoparticle-based antimicrobials are a convenient way to treat MDR pathogens, the implementation of nanoparticles is hindered by several challenges, which include limited knowledge about their particle morphology, toxicity, and difficulties in precise measurement. Furthermore, *in vivo*, the biological barriers complicate their translation into clinical settings^[6]^.

The global crisis of antimicrobial resistance has led to an increase in deaths by at least 50 000 yearly, and it has made the management of OIs, such as nocardiosis, more complicated^[1]^. In this scenario, novel approaches, particularly nanoparticle-based antimicrobials, are considered an alluring and effective method to bypass the infiltration of pathogens resistant to multiple drugs, among the diverse *Nocardia* species with different susceptibility levels depending on the location^[1]^. The nanoparticles’ unique physicochemical properties, such as enhanced membrane disruption, reactive oxygen species generation, and targeted drug delivery^[4,5]^, are being utilized in these innovations, which may greatly reduce the need for and reliance on prolonged and often unsuccessful conventional treatment regimens for immunocompromised patients, thus building on the very limited successes of agents like TMP–SMX and closing the gap in current research that tends to focus on traditional antibiotics while not fully considering nanobiotechnological alternatives^[1,3]^. The field’s progress rests on the comprehensive studies of nanoparticle morphology and toxicity that will help us to overcome the translational barriers^[6]^. In addition, it will be easier to realize the incorporation of *Nocardia*-tailored liposomal amikacin or ciprofloxacin analogs into standard care via the accelerated clinical trials route^[6]^. Last, interdisciplinary collaborations will be the backbone of the geographically adaptive strategies that will eventually tackle the heterogeneous distribution and resistance patterns of *Nocardia*; thus, the broader antimicrobial resistance epidemic will be controlled^[1]^.

In summary, nanobiotechnology is an efficient approach to treating the infection, which is notoriously hard to treat with standard antibiotics. Nanoparticle-based antimicrobials are a considerable strategy for treating multidrug-resistant *Nocardia* infections, despite their divergence in the distribution of *Nocardia* among geographical regions. Moreover, nanobiotechnology should be studied to create efficient strategies to solve the worldwide problem of antimicrobial-resistant bacteria.

## Data Availability

Not applicable to this study.
